# Single-Cell RNA-Seq of Mouse Olfactory Bulb Reveals Cellular Heterogeneity and Activity-Dependent Molecular Census of Adult-Born Neurons

**DOI:** 10.1016/j.celrep.2018.11.034

**Published:** 2018-12-04

**Authors:** Burak Tepe, Matthew C. Hill, Brandon T. Pekarek, Patrick J. Hunt, Thomas J. Martin, James F. Martin, Benjamin R. Arenkiel

**Affiliations:** 1 Program in Developmental Biology, Baylor College of Medicine, Houston, TX 77030, USA; 2 Department of Molecular and Human Genetics, Baylor College of Medicine, Houston, TX 77030, USA; 3 Medical Scientist Training Program, Baylor College of Medicine, Houston, TX 77030, USA; 4 Department of Molecular Physiology and Biophysics, Baylor College of Medicine, Houston, TX 77030, USA; 5 The Texas Heart Institute, 6770 Bertner Avenue, Houston, TX 77030, USA; 6 Cardiovascular Research Institute, Baylor College of Medicine, Houston, TX 77030, USA; 7 Department of Neuroscience, Baylor College of Medicine, Houston, TX 77030, USA; 8 Jan and Dan Duncan Neurological Research Institute at Texas Children’s Hospital, Houston, TX 77030, USA; 9 McNair Medical Institute, Baylor College of Medicine, Houston, TX 77030, USA; 10 These authors contributed equally; 11 Lead Contact

## Abstract

Cellular heterogeneity within the mammalian brain poses a challenge toward understanding its complex functions. Within the olfactory bulb, odor information is processed by subtypes of inhibitory interneurons whose heterogeneity and functionality are influenced by ongoing adult neurogenesis. To investigate this cellular heterogeneity and better understand adult-born neuron development, we utilized single-cell RNA sequencing and computational modeling to reveal diverse and transcriptionally distinct neuronal and nonneuronal cell types. We also analyzed molecular changes during adult-born interneuron maturation and uncovered developmental programs within their gene expression profiles. Finally, we identified that distinct neuronal subtypes are differentially affected by sensory experience. Together, these data provide a transcriptome-based foundation for investigating subtype-specific neuronal function in the olfactory bulb (OB), charting the molecular profiles that arise during the maturation and integration of adult-born neurons and how they dynamically change in an activity-dependent manner.

## In Brief

Using single-cell sequencing, Tepe et al. describe cellular heterogeneity in the mouse olfactory bulb, uncover markers for each cell type, and reveal differentially regulated genes in adult-born neurons. These findings provide a framework for studying cell-type-specific functions and circuit integration in the mammalian brain.

## Graphical Aabstract

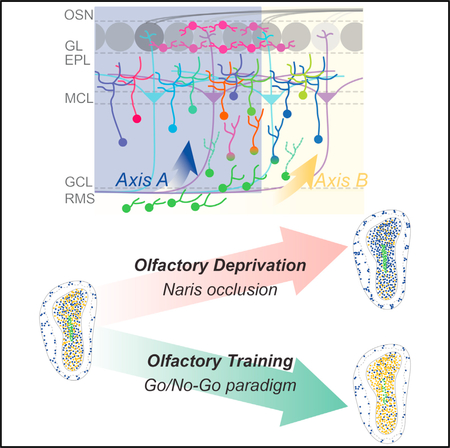

## INTRODUCTION

A fundamental challenge in understanding brain function is our limited knowledge of the cellular heterogeneity in the brain. Recent advances in single-cell RNA sequencing allow molecular profiling of individual cells from large and intermingled popula tions (Ziegenhain et al., 2017). Importantly, profiling populations of neuronal and nonneuronal cells is beginning to unveil the rich cellular heterogeneity that comprises different brain systems and offers insight into how this cellular heterogeneity contributes to function. Additionally, defining and profiling cellular subtypes yields unique markers that can be used to identify and manipulate targeted cell types. As cell-type-specific manipulations become increasingly important for determining neuronal circuit function, revealing molecular profiles for cellular subtypes provides an invaluable resource.

Sensory processing and perception is a fundamental brain function. Olfaction is a crucial sensory modality that many species depend on for survival, social interaction, feeding, and mating. In mammals, olfactory sensory neurons (OSNs) receive odor information from the environment, and relay it to the olfactory bulb (OB) (Buck, 1996; Shepherd, 1994). Each OSN projects to specific glomeruli based on odorant receptor expression. OSNs expressing the same receptor converge onto the same glomeruli, where they synapse with excitatory mitral and tufted (M/T) cells (Mombaerts et al., 1996; Ressler et al., 1994; Sakano, 2010; Vassar et al., 1994). M/T cells project to deeper brain regions for further olfactory sensory processing (Lepousez and Lledo, 2013; Mori and Sakano, 2011; Mori et al., 1999). However, within the olfactory bulb, M/T cell activity is shaped by local inhibitory interneurons (Abraham et al., 2010; Tan et al., 2010). Olfactory bulb interneuron populations include diverse cell types, with the most abundant being granule cells (GCs) (Burton, 2017; Lledo et al., 2008). Together, granule cells far outnumber other O olfactory bulb B interneurons, but differences in granule cell morphology, anatomical location, and electrophysiological properties suggest a substantial molecular heterogeneity within this population (Carleton et al., 2003; Merkle et al., 2007, 2014). Thus, deciphering the different subtypes of interneurons that make up the olfactory bulb and investigating their contributions toward olfactory bulb circuit function are essential for understanding olfaction. Although existing markers allow for genetic labeling and manipulation of broad olfactory bulb interneuron classes, molecular signatures of finer subtypes remain unknown, and it is likely that distinct interneuron sub-types have yet to be identified.

A potential source of cellular diversity in the olfactory bulb is ongoing adult neurogenesis (Alvarez-Buylla and Lim, 2004; Gage, 2000; Lledo et al., 2008). Adult-born neurons originate from the subventricular zone (SVZ) of the lateral ventricles (Merkle et al., 2004) and migrate anteriorly, ultimately integrating into existing olfactory bulb circuits (Ming and Song, 2011). This population of adult-born neurons become inhibitory inter-neurons, primarily differentiating into granule cells and periglomerular cells (PGCs) (Carleton et al., 2003; Lledo et al., 2006). Throughout the process of maturation and integration, roughly half of all adult-born neurons are eliminated via apoptosis, while the rest integrate into existing circuitry (Ryu et al., 2016). Interestingly, this fate decision is dependent upon the levels of circuit activity received during synapse formation and circuit integration (Henegar and Maruniak, 1991; Murray and Calof, 1999). While olfactory deprivation by naris occlusion reduces the survival of integrating neurons into the olfactory bulb (Mandairon et al., 2006; Yamaguchi and Mori, 2005), olfactory learning promotes survival and integration (Alonso et al., 2012; Mouret et al., 2008; Quast et al., 2017; Rochefort and Lledo, 2005; Rochefort et al., 2002). Thus, olfactory experience directly influences the integration of adult-born interneurons into olfactory bulb circuitry, though the molecular mechanisms driving activity-dependent circuit integration are not fully understood.

To develop a comprehensive profile of cellular heterogeneity within the olfactory bulb, and to investigate how sensory activity affects the molecular programs that promote the survival and integration of adult-born neurons, we employed high-throughput single-cell RNA sequencing (scRNA-seq) (Zheng et al., 2017) of wild-type and activity manipulated mouse olfactory bulbs. This technique allowed an in-depth categorization of single cell molecular signatures, and revealed developmental and activity-dependent changes that occur in adult-born neuron populations. Together, our data inform the heterogeneity of interneurons within the olfactory bulb, provide a molecular blueprint of developmental programs for adult-born neurons, and reveal the transcriptional changes that govern their activity-dependent circuit integration. Furthermore, our results suggest that distinct molecular mechanisms act on different subsets of adult-born neurons, driving diversity and survival of adult-born interneuron subsets in an activity-dependent manner.

## RESULTS

### Single-Cell Sequencing Establishes a Molecular Census of Olfactory Bulb Cells

**Figure 1.**
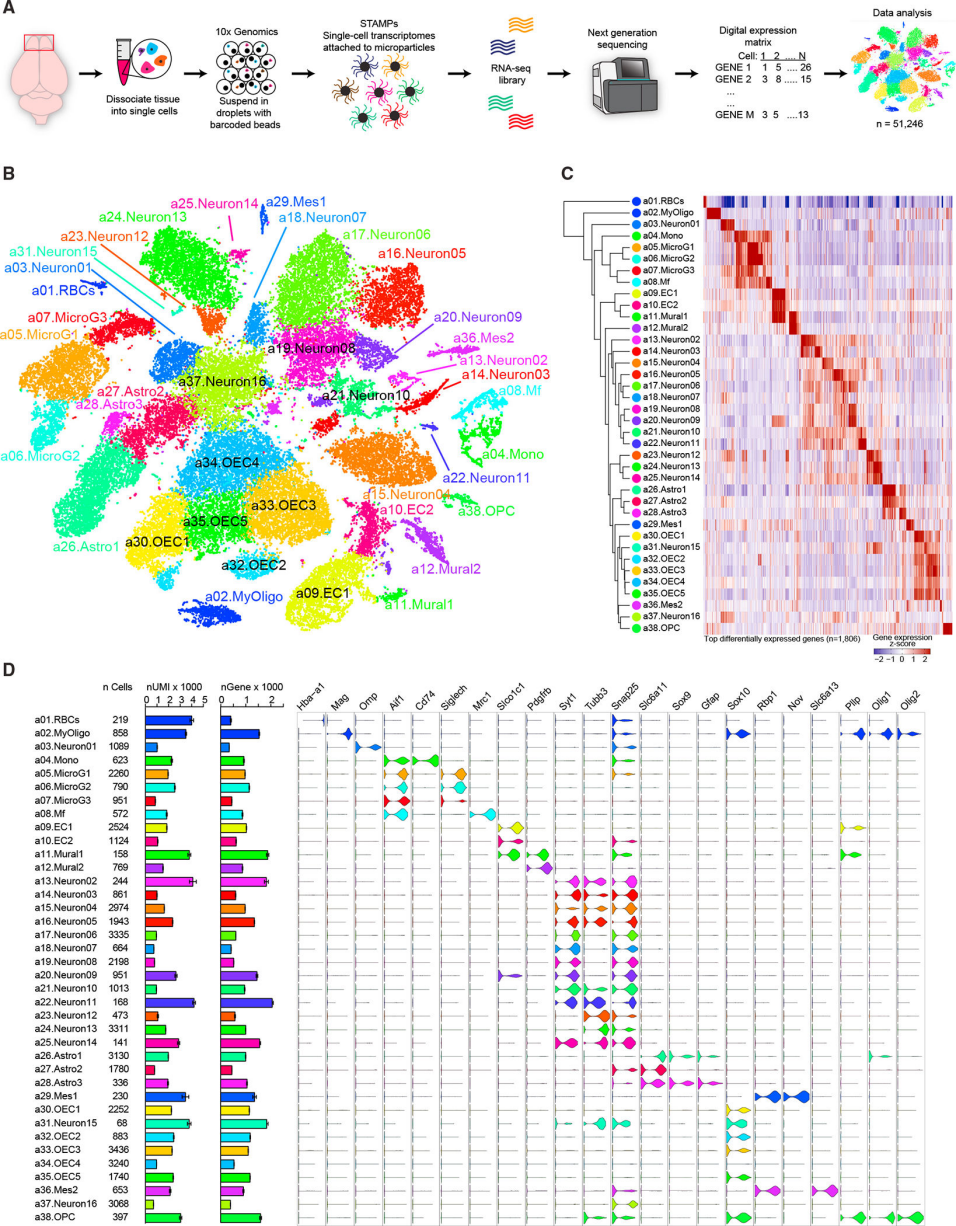
Single-Cell Transcriptome Analysis Delineates Mouse Olfactory Bulb Cellular Heterogeneity (A) Schematic view of the experimental workflow. (B) Cellular composition of the olfactory bulb was visualized using t-distributed stochastic neighbor embedding (t-SNE). Individual single-cell transcriptomes were colored according to cluster identity in (B)–(D). (C) Dendrogram depicting hierarchical relationships between distinct cell populations. Heatmap illustrating the genes most highly enriched in each cluster, with each column representing a gene and each row representing average expression level of that gene in each cluster. (D) Graph showing number of cells per cluster, number of unique molecular identifiers (UMIs) per cluster (mean ± SEM; scale is in thousands), and number of genes detected per cluster (mean ± SEM; scale is in thousands). Violin plots show expression of cell-type-specific marker genes for each cluster. See also Figure S1 and Table S1.

To elucidate the overall cellular heterogeneity and activity dependent changes in olfactory bulb composition, we profiled the transcriptomes of 51,246 single cells collected from the olfactory bulbs of wild-type adult mice (Figure 1A). Mice were naive, olfactory deprived through naris occlusion, or olfactory enriched through training on an olfactory-discrimination learning paradigm. To block olfactory sensory input, we performed unilateral naris occlusion, with the occluded side serving as the sensory-deprived sample and the open side as the control (Najbauer and Leon, 1995; Quast et al., 2017; Yamaguchi and Mori, 2005). Mice were trained to discriminate various odorants using an olfactory-cued learning paradigm (Liu et al., 2017; Liu et al., 2018). This form of olfactory training exposed mice to several different odorants while also actively engaging the olfactory system to facilitate olfactory-discrimination learning. Cells from naive, olfactory-deprived, and enriched mice were clustered together after single-cell sequencing based on similarities in their transcriptional profiles using an unsupervised principal-component analysis (Macosko et al., 2015) and visualized using t-distributed stochastic neighbor embedding (t-SNE) (Van der Maaten, 2014; Van Der Maaten and Hinton, 2008) (Figure 1B). We identified 38 distinct cellular clusters, each composed of cells from different olfactory experience paradigms, indicating that experimental conditions did not bias the identity of the clusters (Figure S1). Using the expression patterns of cluster-enriched genes, we next assigned identities to each cluster. In total, we observed 16 neuronal (*Syt1*^+^/*Tubb3*^+^), three astrocytic (*Gfap*^+^), five olfactory ensheathing cell-based (*Sox10*^+^), six hematopoietic (all *Aif1*^+^; three *Siglich*^+^ microglia, one *CD52*^+^ macrophage, one *CD74*^+^ monocyte, and one *Hba a1*^+^ red blood cell), four blood-vessel-based (two *Slco1c1*^+^ endothelial and two *Pdgfrb*^+^ mural), one oligodendrocyte-precursor-based (*Olig2*^+^), one myelinating-oligodendrocyte-based (*Mag*^+^), and two mesenchymal clusters (Figures 1B and 1D). Together, these data reveal the overall transcriptional heterogeneity of the cell types that comprise the mammalian olfactory bulb and identify molecular markers to further investigate the diversity and function of olfactory bulb cell types.

### Transcriptome-Based Clustering of Neurons Identifies Markers of Neuronal Subtypes

**Figure 2.**
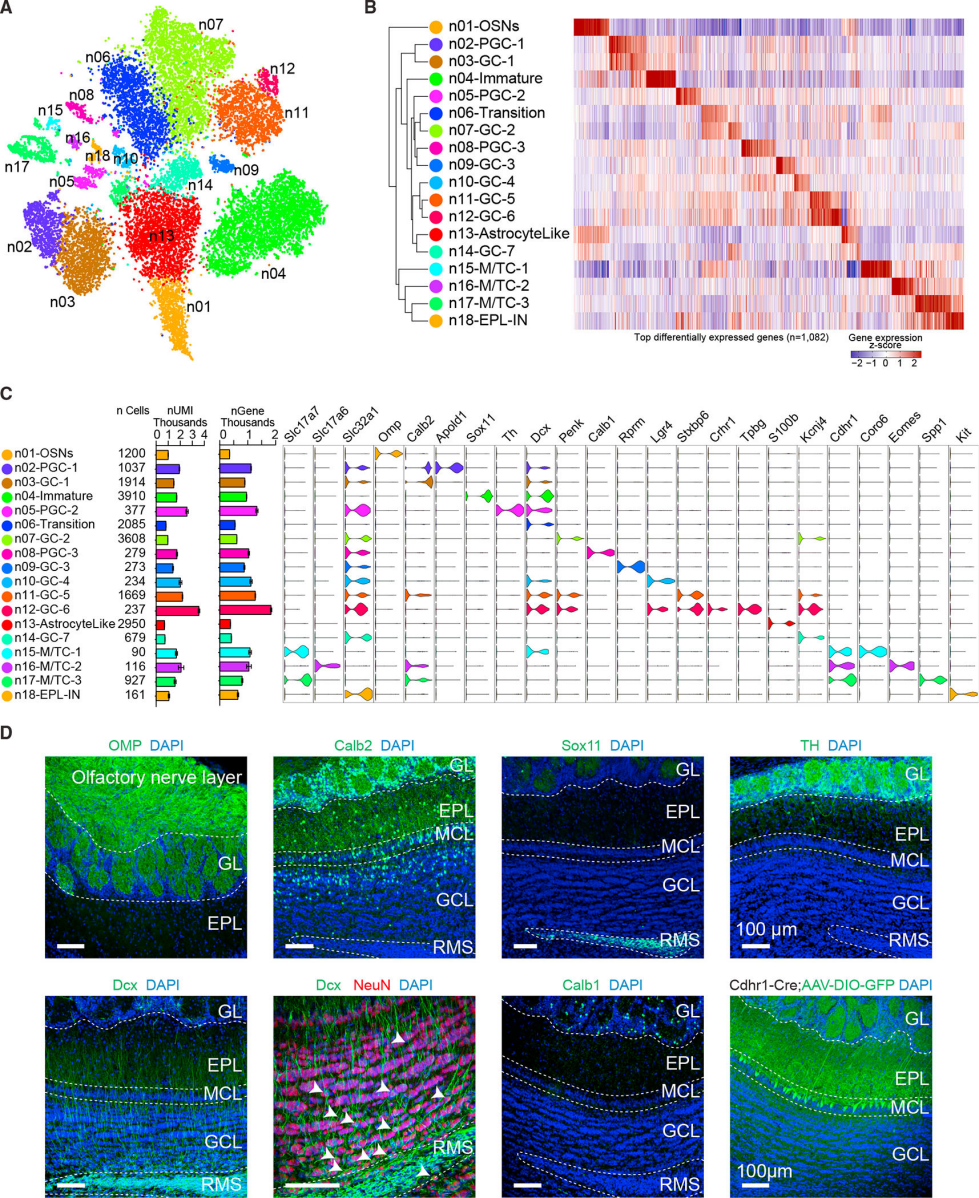
Unsupervised Transcriptome-Based Clustering Reveals Neuronal Subtypes (A) Neuronal subclusters were visualized using t-SNE. Individual single-cell transcriptomes were colored according to cluster identity in (A)–(C). (B) Dendrogram depicting hierarchical relationships between distinct neuronal cell types. Heatmap illustrating the genes most highly enriched in each cluster, with each column representing a gene, and each row representing the average expression level of that gene in each neuronal cluster. (C) Graph showing predicted neuronal class of each cluster (EPL-IN, external plexiform layer interneuron; GC, granule cell; M/TC, mitral and tufted cell; OSN, olfactory sensory neuron; PGC, periglomerular cell), number of cells per cluster, number of UMIs per cluster (mean ± SEM; scale is in thousands), and number of genes detected per cluster (mean ± SEM; scale is in thousands). Violin plots showing expression of neuronal cell-type-specific marker genes for each cluster. (D) Immunohistochemistry against known cell-type-specific markers that label different neuronal clusters. Calb1, calbindin 1; Calb2, calbindin 2; *Cdhr1-Cre; cadherin 1-Cre* mouse injected with a conditional AAV reporter labeling M/T cells; Dcx, doublecortin; NeuN, neuronal nuclei; OMP, olfactory marker protein; Sox11, sex-determining region Y-box 11; TH, tyrosine hydroxylase. Scale bars, 100 um. See also Figure S2 and Table S2.

To assign specific identities to neuronal subtypes within the olfactory bulb, we filtered and subclustered neurons identified from initial clustering (Figure 1B, Neuron01–Neuron16). Neurons were initially identified utilizing gene enrichment data (Figure 1C; Table S1) by selective expression of known neuronal markers such as *Syt1* and *Tubb3.* We next subclustered neurons based on similarities of their transcriptional profiles using an unsupervised principal-component analysis similar to whole data analysis and visualized their distribution through t-SNE (Figure 2A). Using the expression patterns of cluster-enriched marker genes, we determined that one cluster represented OSNs (n01-OSNs), three represented M/T cells (n15-M/TC-1, n16-M/TC-2, and n17-M/TC-3), and the remaining 14 represent mature and immature inhibitory neurons (Figure 2). To further classify and validate cluster identities, we examined the expression of known neuronal subtype markers, cross-referenced and examined RNA *in situ* hybridization (ISH) expression profiles from the Allen Brain Atlas (Lein et al., 2007), and performed immunohistochemical analysis (Figures 2D and S2A).

Notably, we observed that genes enriched in different clusters differentially labeled distinct layers and cell types within the olfactory bulb (Figure S2A). For example, genes enriched in clusters n15-M/TC-1, n16-M/TC-2, and n17-M/TC-3 strongly labeled cells in the mitral cell layer (MCL) and expressed the known M/T cell marker *Cdhr1* (Nagai et al., 2005) (Figures 2C, 2D, and S2A). Cluster n16-M/TC-2 expressed the transcription factor *Tbr2* (Eomes), which marks excitatory cells located in the periglomerular layer (Brill et al., 2009). Genes enriched in cluster n18-EPL-IN labeled the external plexiform layer (EPL), which comprises EPL interneurons. Cells in clusters n05-PGC-2 and n08-PGC-3 expressed interneuron markers (*Th*+ and *Calb1*+, respectively) that localize to periglomerular regions, suggesting that these clusters represent two distinct subtypes of inhibitory periglomerular cells (Kosaka and Kosaka, 2005; Toida et al., 2000). Finally, and to our direct interest, genes enriched in clusters n03-GC-1, n06-Transition, n07-GC-2, n09-GC-3, n10-GC-4, n11-GC-5, n12-GC-6, and n14-GC-7 labeled cells within the granule cell layer, indicating distinct subsets of granule cells. Although we could not identify genes uniquely labeling some clusters (such as n06-Transition and n14-GC-7), based on genes enriched in these clusters (such as *Dcx* in n06-Transition and *Slc32a1* in n14-GC-7), we concluded that they likely represent immature and mature clusters of adult-born neurons, respectively. It has been previously shown to be challenging to identify unique markers for single cellular clusters; rather, combinatorial expression patterns of multiple markers should be considered to differentiate cellular clusters (Li et al., 2017).

*Slc32a1* is known to be expressed in mature inhibitory inter-neurons, and we observed expression in several neuronal clusters, consistent with the idea that the olfactory bulb is heavily populated by inhibitory interneurons. Clusters not expressing *Slc32a1* represented either excitatory clusters (n01-OSNs, n15-M/TC-1, n16-M/TC-2, and n17-M/TC-3) or developing immature cell clusters (n04-Immature and n06-Transition). Adult neurogenesis contributes to the diversity of the observed granule cell clusters, and given that adult-born neurons exist at multiple stages of development, these data suggested that some clusters represented transitional stages during adult-born neuron integration. For example, *Tpbg* (5T4) labeled cluster n12-GC-6 and localized to superficial regions of the granule cell layer, suggesting that these cells are mature adult-born granule cells. In support of this, *Tpbg*+ granule cells have been previously shown to be required for proper odor detection and discrimination (Takahashi et al., 2016), and these same cells also express *Crhr1,* which we have previously reported to label mature granule cells and regulate circuit integration and synapse formation in adult-born neurons (Garcia et al., 2014, 2016). Lastly, genes enriched in cluster n04-Immature (such as *Sox11*) labeled cells residing in the rostral migratory stream, indicating that these represent immature migrating adult-born neurons (Figures 2D and S2A). Cluster n04-Immature was also highly enriched in *Dcx* expression, which is a direct regulator of neuronal migration in both embryonic and adult neurogenesis (Francis et al., 1999; Gleeson et al., 1999). We also observed *Dcx*-positive cells in other putative mature clusters, which may be explained by the perdurance of *Dcx* transcription throughout migration and maturation. To investigate this, we performed immunohistochemistry on olfactory bulb sections against the mature neuronal marker NeuN and the immature neuron marker Dcx and observed cells expressing both (Figure 2D). Thus, our data reveal subclusters of diverse neurons and glia within the olfactory bulb, which have been delineated by their differential transcriptional profiles. Together, these profiles provide a transcriptomic map to facilitate cell-type-specific identification, labeling, and manipulation of mouse olfactory bulb cell types.

### Pseudo-Timeline Analysis Reveals Transcriptional Changes throughout Adult-Born Neuron Maturation

**Figure 3.**
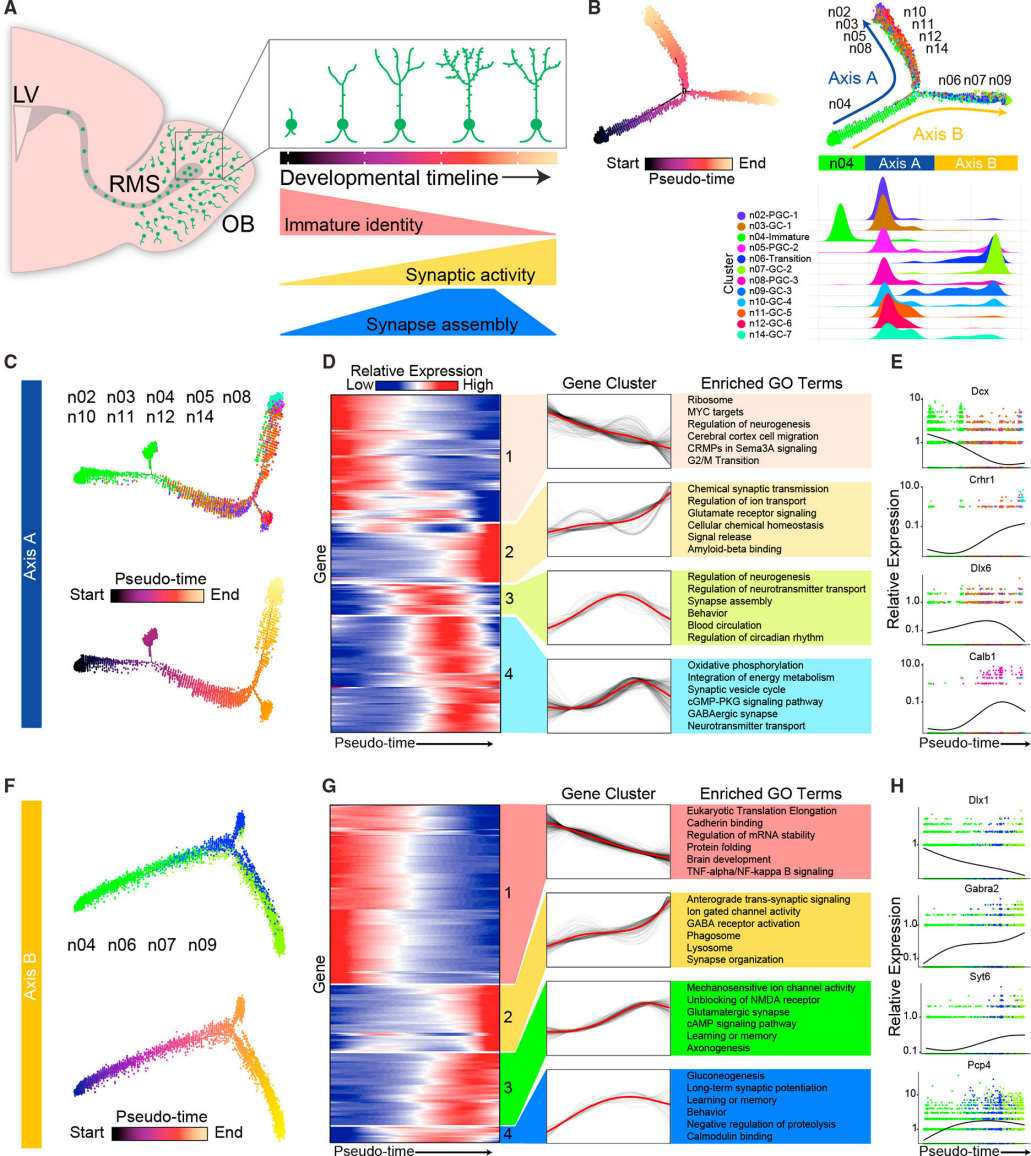
Pseudo-Timeline Analysis Reveals Transcriptional Changes during Maturation and Integration along Distinct Developmental Axes (A) Schematic sagittal view of the mouse olfactory system. Inset: summary diagram of olfactory bulb adult-neurogenesis illustrating broad morphological and developmental changes throughout maturation of adult-born neurons. LV, lateral ventricle; RMS, rostral migratory stream. (B) (Top left) Monocle2 pseudotime trajectory of adult-born neurons. Cells are colored by pseudotime score, with dark colors representing immature cell stages and light colors representing mature cell stages. (Top right) Monocle2 pseudotime trajectory of adult-born neurons with cells colored by cluster identity according to Figure 2. (Bottom) Adult-born neuron cluster density plot projected to the x axis of the bifurcating Monocle2 pseudotime trajectory, indicating which arm of the timeline each cell type is located. (C) (Top) Axis A of Monocle2 pseudotime trajectory colored according to cluster identity. (Bottom) Axis A pseudotime trajectory colored by pseudotime score, with the dark color representing an immature cell stage and the light color representing a mature cell stage. (D) Axis A: 4 distinct groups of pseudotime-dependent genes with dynamic expression patterns plotted across pseudotime as heatmaps, with blue indicating low levels and red indicating high levels of expression. (Middle) Gene expression trends for each gene (black) with the trend line highlighted in red. (Right) Top 6 enriched gene ontology (GO) terms for each temporal cluster. (E) Differential expression patterns of one example gene from each group of genes along developmental axis A. (F) (Top) Axis B of Monocle2 pseudotime trajectory colored according to cluster identity. (Bottom) Axis B pseudotime trajectory colored by pseudotime score, with the dark color representing an immature cell stage and the light color representing a mature cell stage. (G) Axis B: 4 distinct groups of pseudotime-dependent genes, with dynamic expression patterns plotted across pseudotime as heatmaps. Blue indicates low levels and red indicates high levels of expression. (Middle) Gene expression trends for each gene (black), with the trend line highlighted in red. (Right) Top 6 enriched gene ontology (GO) terms for each temporal cluster. (H) Differential expression patterns of one example gene from each group of genes along developmental axis B. See also Tables S3 and S4.

To investigate common molecular mechanisms that guide adult-born neuron circuit integration, we next examined transcriptional changes in adult-born neuron subclusters throughout their developmental progression. Ongoing neurogenesis in the olfactory bulb provides snapshots of development at different stages. At any given time, thousands of adult-born neurons at various developmental stages are migrating to the olfactory bulb and integrating into circuits (Ming and Song, 2005). Since the generation and integration of these cells is asynchronous, we sought to examine the differential stages of their integration (Figure 3A). To this end, we implemented the program Monocle2 to identify and order developing adult-born neurons in “pseudotime” (Trapnell et al., 2014). Monocle2 computationally orders cells in an unsupervised manner by maximizing the transcriptional similarity between successive pairs of cells. Thus, this approach can be used to define different maturation stages of adult-born neurons. To increase the specificity of this analysis, we excluded all non-adult-born neuronal populations, including those expressing markers of M/T cells (n15-M/TC-1, n16-M/TC-2, and n17-M/TC-3), EPL interneurons (n18-EPL-IN), OSNs (n01-OSNs), and ambiguous neuronal cell types (n13-AstrocyteLike). Cells arising from adult-born neuronal lineages were then ordered along a putative developmental trajectory (pseudo-timeline) from least to most differentiated (Figure 3B). Interestingly, ordering all clusters that constitute adult-born lineages revealed a bifurcation of the pseudo-timeline, suggesting that distinct molecular pathways guide the development of different populations.

To further characterize the bifurcated trajectories, we determined the cluster composition of each branch (Figure 3B, right panel), and divided our analysis into two axes (Figures 3C–3F). Plotting cluster identities onto the bifurcated timeline revealed that cluster n04 contained the most immature cells, which was supported by their expression of immature neuronal markers (Table S2). Additionally, we found that clusters n02-PGC-1, n03-GC-1, n05-PGC-2, n08-PGC-3, n10-GC-4, n11-GC5, n12-GC-6, and n14-GC-7 were positioned on axis A, and clusters n06-Transition, n07-GC-2 and n09-GC-3 were located on axis B (Figure 3B, right panel). To determine which genes regulate the progression of adult-born neurons along each axis, we performed hierarchical clustering of the genes whose expression varied as a function of pseudotime. In support of the plotted pseudo-timeline order, our data revealed groups of genes that showed differential expression along each axis, which correlated well with genes that are known to be developmentally regulated throughout adult-born neuron maturation and integration, such as *Dcx*/*Dlx* family transcription factors, and mature neuronal markers, such as *Crhr1* and *Calb1* (Figures 3D, 3E, 3G, and 3H) (Garcia et al., 2014, 2016; Ming and Song, 2011). Importantly, gene ontology (GO) analysis showed that both axes A and B were highly enriched for genes involved in developmental processes and that genes involved in early neuronal development are downregulated as the pseudo-timeline progresses on both axes (Figures 3D and G). Further corroborating the validity of pseudo-timeline analysis, genes with increased expression along the timeline included those involved in synapse formation and function and programs inherent to later stages of neuronal development. Interestingly, a subset of genes that were transiently upregulated and then downregulated have been implicated in synaptic assembly and long-term potentiation (LTP), such as *Pcp4*, *CaMKII*, and *MAPK1* (Lisman et al., 2002; Peng et al., 2010; Wei et al., 2011). Adult-born olfactory bulb neurons display high plasticity early in development and lose such plasticity as they terminally differentiate into resident granule cells (Nissant et al., 2009). These data support this and further reveal genes that may regulate and/or participate in aspects of synaptic plasticity and neural circuit formation (Table S3). Moreover, axis A was enriched in genes that regulate overall cellular metabolism and neuronal morphogenesis, whereas axis B was enriched for genes that regulate RNA processing and cell adhesion. Such transcriptional differences indicate that the development of these two different olfactory bulb interneuron populations may be guided by distinct molecular pathways. Together these data and analyses reveal a clear developmental staging of adult-born neurons in the olfactory bulb, whereby differential gene expression along the bifurcated timeline indicates that each axis utilizes distinct pathways for neuronal maturation.

### Gene Regulatory Networks Mark Stable Stages of Adult-Born Neuron Development

Neuronal identity and cell fate are governed by transcription factors and their associated cofactors, working together to regulate target gene expression typical of given cell types. The collection of interacting transcription factors and cofactors that govern gene expression, and thus identity, can be referred to as a gene regulatory network (GRN). Cataloging functional GRNs and their temporal dynamics during adult-born neuronal migration, maturation, integration, and elimination allows for a better understanding of the molecular mechanisms that drive these processes. With this goal in mind, we deployed a single-cell regulatory network inference and clustering (SCENIC) computational pipeline to map GRNs in adult-born olfactory bulb neurons (Aibar et al., 2017).

**Figure 4.**
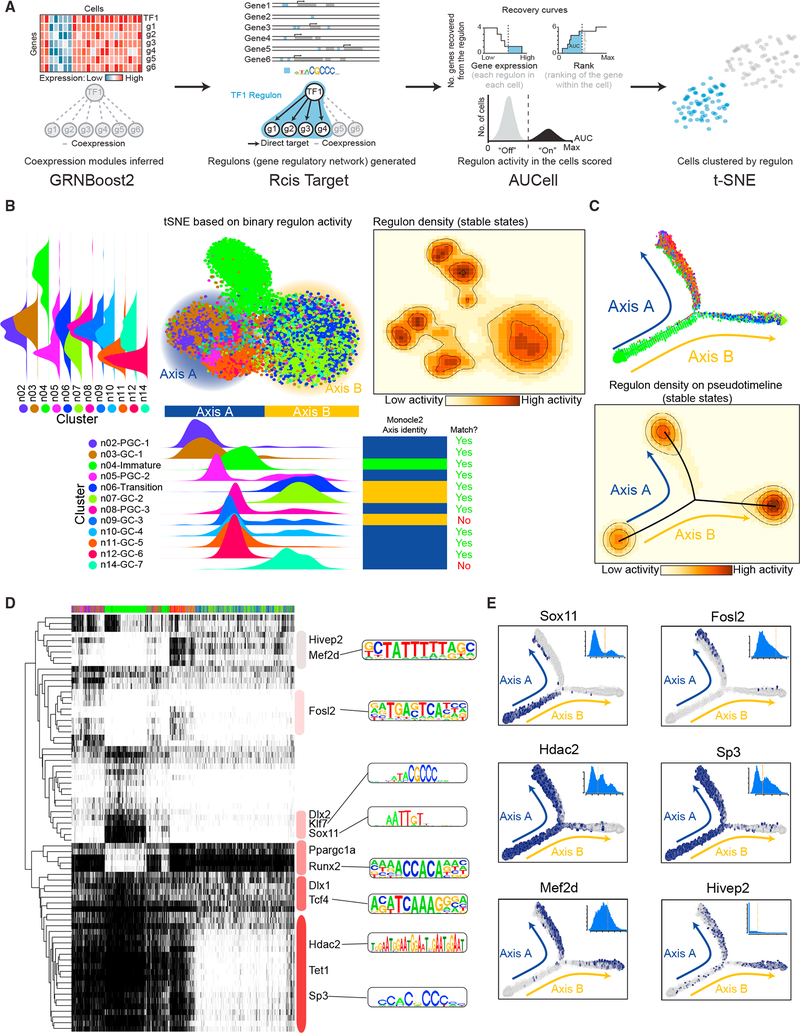
Gene Regulatory Networks Mark Stable Stages of Adult-Born Neuron Development (A) Schematic view of the SCENIC workflow, as described in the results. (B) (Top left) Adult-born neuron cluster density plot calculated from the binary regulon activity-based t-SNE projected on the y axis of the graph. (Top center) Adult-born neurons visualized using t-SNE based on the binary adult-born neuron regulon activity matrix. Clusters were colored according to adult-born neuron cluster identity from initial clustering in Figure 2. (Top right) Regulon activity density plotted on regulon-based t-SNE. Light colors represent low regulon activity, and darker colors represent high regulon activity. (Bottom left) Adult-born neuron cluster density plot calculated from the binary regulon activity-based t-SNE projected on the x axis of the graph. (Bottom right) Monocle2 axis identity for each cell cluster (blue, axis A; yellow, axis B; green, immature cluster) and whether the identity matched the regulon-based clustering. (C) (Top) Adult-born neuron Monocle2 pseudotime trajectory colored according to adult-born neuron cluster identity. (Bottom) Regulon activity density plotted on the adult-born neuron Monocle2 pseudotime trajectory calculated in Figure 3. Lighter colors represent low regulon activity, and darker colors represent high regulon activity (D) SCENIC binary regulon activity matrix showing all correlated regulons (absolute correlation >0.3) that were active in at least 1% of all adult-born neurons. Each column represents a single cell, colored according to cluster identity. Representative transcription factors are highlighted along with corresponding DNA-binding motifs on the right side of the matrix. (E) Adult-born neuron Monocle2 pseudo-timeline, colored according to the corresponding SCENIC binary regulon activity; blue indicates active regulons, and gray indicates inactive regulons. The inset histograms denote the AUCell score distribution for the regulon.

Within the SCENIC framework, we identified genes co-expressed with certain transcription factors using the GRNboost2 fast GRN inferencing algorithm (Friedman, 2002). Next, we performed *cis*-regulatory motif enrichment analysis on all co-expressed genes. This analysis cataloged putative transcription-factor-binding sites within the list of co-expressed genes, thereby allowing us to identify potential direct gene targets. Additionally, this allowed us to reduce false positives and indirect transcriptional targets from the co-expression matrix. Transcription factors typically regulate several genes. Genes regulated by a specific transcription factor, identified from motif enrichment analysis, were grouped together into units referred to as regulons. A regulon thus represents a list of putative target genes for a particular transcription factor. Finally, to calculate the activity of each regulon in single-cell transcriptomes, we applied a SCENIC AUCell algorithm (Aibar et al., 2017), whereby individual cells that express the greatest number of associated genes within a given regulon display the highest area under the curve (AUC) score, while those expressing fewer regulon genes receive lower AUC scores. The ranked distribution of AUCell scores across cells for a given regulon was used to determine a threshold for active and inactive regulons, thus making the final output binary (active or inactive). Through this analysis, we identified 299 regulons that were active in adult-born neurons out of 698 initially present in our transcription factor co-expression matrix. To compare the results of SCENIC with our previous expression-based clustering analysis (Figure 2) and Monocle2 pseudo-timeline results (Figure 3), we performed t-SNE using the binary regulon activity calculated by the SCENIC pipeline (Figure 4B, top panel). The resulting t-SNE revealed a bifurcated distribution of adult-born interneuron clusters that closely matched both expression-based t-SNE (Figure 2A) and Monocle2 pseudo-timeline results (Figure 3B). The only clusters the data did not fully match were n09-GC-3 and n14-GC-7. This may be due to the large number of genes utilized for Monocle2 pseudo-timeline analysis compared to the limited number of regulons used for gene regulatory network analysis. Interestingly, however, regulon density plotted on regulon based clustering (higher regulon activity is indicated by the darker color) revealed three distinct cell states (Figure 4B, right panel). Plotting regulon activity density on the Monocle2 pseudo-timeline revealed that these three stable cell states reside at the beginning of the pseudo-timeline and at the end of each developmental axis (Figure 4C). This finding is consistent with a model in which cells between these points are differentiating and thus are likely to be less transcriptionally stable (Wu et al., 2010).

To characterize temporal GRN dynamics during adult-born neuronal maturation, we next assessed regulon activity across the Monocle2 pseudo-timeline (Figures 4C–4E). Consistent with interneuron identity, nearly all analyzed adult-born neurons showed high *Dlx1*, *Runx2*, and *Tcf4* regulon activity (Figure 4D) (Cobos et al., 2005; Subburaju and Benes, 2012). The most immature cell state, composed mainly of cluster n04, showed high *Dlx2*, *Klf7*, and *Sox11* activity—known markers for neurogenesis (Laub et al., 2005; Sellers et al., 2014; Wang et al., 2013). Axis A, however, showed activity for several regulons likely representing more mature states, including *Hdac2*, *Fosl2*, and *Sp3* (Figure 4E). These factors have recently been found to form a functional protein complex that represses target gene expression, ultimately decreasing synaptic plasticity (Yamakawa et al., 2017). Interestingly, *Sp3* function has also been associated with induction of programmed cell death (Essafi-Benkhadir et al., 2009). Cells along axis B showed high levels of a diverse array of regulons, including *Hivep2* and *Mef2d* (Figure 4E), which have recently been implicated in synaptic regulation and neuronal survival (Chan et al., 2014; Harrington et al., 2016; Mayer et al., 2018). Overall, GRN analysis revealed that distinct regulons label different axes of adult-born neuron development, suggesting that such regulons may guide maturation to distinct molecular programs during olfactory bulb neurogenesis and dictate terminal fate acquisition.

### Olfactory Sensory Activity Regulates Axis-Specific Adult-Born Neurogenesis

**Figure 5.**
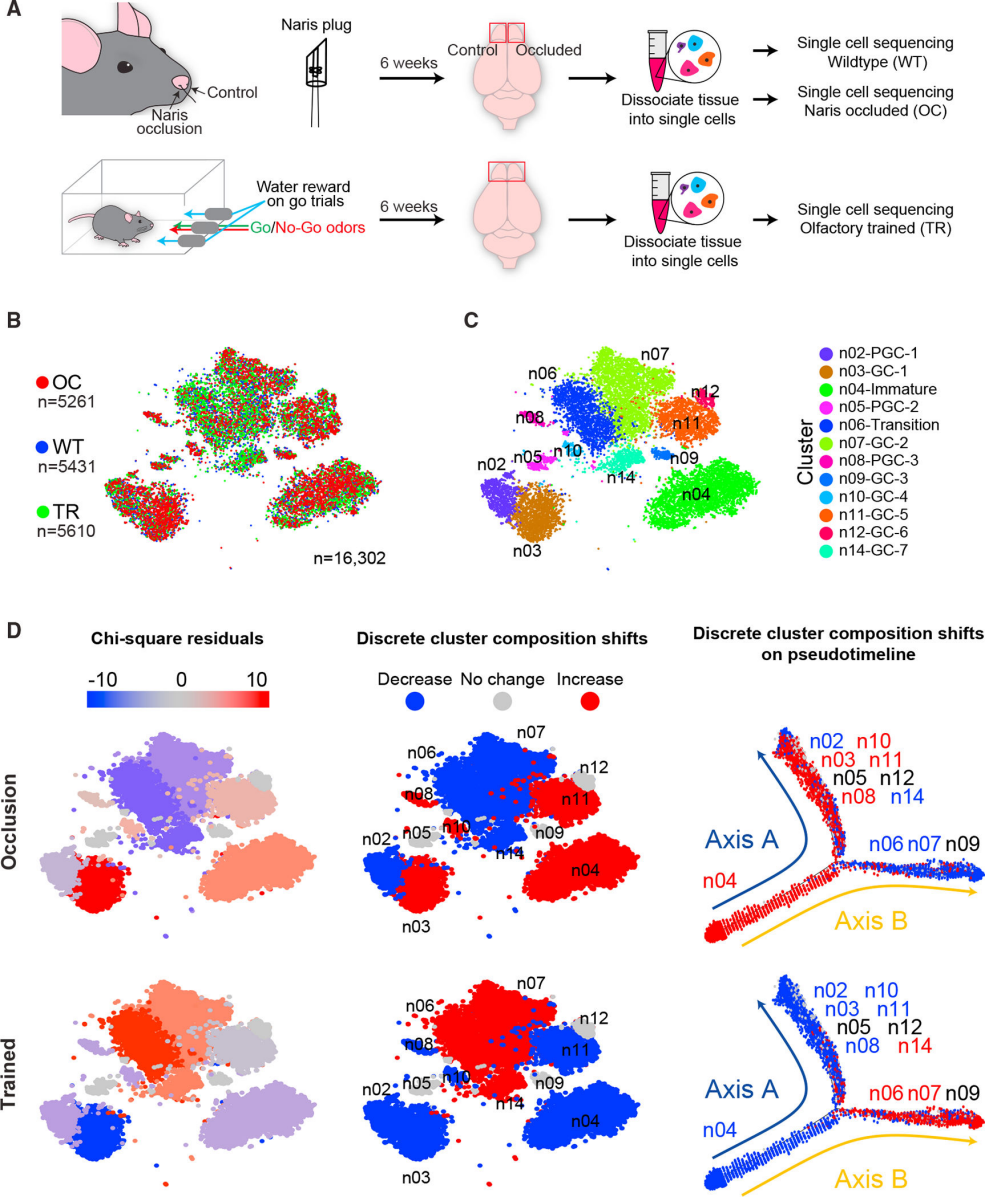
Olfactory Activity Alters Adult-Born Interneuron Subtype Composition (A) Schematic view of olfactory bulb experimental procedures, which are detailed in methods. (B) Two-dimensional t-SNE representation of 16,302 adult-born interneurons colored according to experimental group. OC, naris occluded; TR, olfactory trained; WT, wild-type. (C) t-SNE representation of 16,302 adult-born interneurons colored according to cluster identity. (D) Shifts in adult-born neuron cluster composition for each indicated experimental condition plotted across expression-based t-SNE (left and middle) and along the Monocle2 pseudotime trajectory (right). Pearson’s chi-square test residuals were calculated for the corresponding experimental group (left). Discrete values were determined from Pearson’s chi-square test (middle); if the p value was < 0.05, then clusters were assigned appropriate designation of increased or decreased (red or blue) based on their residual score. Clusters with no significant composition shift are highlighted in gray (no change, p value > 0.05). See also Figure S3.

Previous studies have shown that olfactory sensory deprivation reduces adult-born neuron circuit integration and survival (Mandairon et al., 2006; Yamaguchi and Mori, 2005). Conversely, mice trained to discriminate olfactory stimuli show increased synaptogenesis and survival (Alonso et al., 2012; Mouret et al., 2008; Rochefort and Lledo, 2005; Rochefort et al., 2002). Although these findings have been invaluable in drawing the link between neural activity and adult-born neuron circuit integration, they were not sufficient at the time to resolve cell-type-specific maturation programs. To investigate activity-dependent changes in the cellular composition of olfactory bulb circuits, we analyzed differences between scRNA-seq data from both olfactory-deprived mice and those trained on an olfactory-discrimination task (Figure 5A). For this, we first trained a group of mice. We then performed scRNA-seq on olfactory bulbs from deprived, trained, and naive controls and compared the adult-born lineages in each of the three conditions (Figure 5B). We implemented chi-square tests using an expected equal distribution of cells across all experimental conditions to assess statistically significant changes in cell distribution in each cluster (Figure 5D). We observed that clusters n05-PGC-2 (*Th*+), n09-GC-3 (*Rprm*+), and n12-GC-6 (*Tpbg*+) did not change in response to altered olfactory activity; however, clusters n06-Transition, n07-GC-2, and n14-GC-7 showed significant differences following manipulation of olfactory activity (Figure 5D). Notably, olfactory deprivation reduced the proportions of cells that comprise these clusters, while olfactory enrichment increased the proportion of cells that comprised the clusters (Figure 5D). For clusters n03-GC-1, n04-Immature, n08-PGC-3, n10-GC-4, and n11-GC-5, however, olfactory deprivation increased proportions of cells in the clusters, while olfactory training decreased their overall composition. Interestingly, clusters n06-Transition and n07-GC-2 sorted to one arm of the Monocle2 based pseudo-timeline, while cluster n03-GC-1, n08-PGC-3, n10-GC-4, and n11-GC-5 segregated to the other (Figure 5D right panel).

To test whether these changes were due to differential regulation of adult-born neuron development, or transcriptional state switching within integrated interneurons, we performed a line-age-tracing experiment. To selectively label adult-born neurons both spatially and temporally, we crossed *Dlx1/2-CreER* mice (Batista-Brito et al., 2008) with a *Rosa-LoxP-Stop-LoxPTdTomato* reporter line, which expresses the tdTomato fluores-cent protein after tamoxifen-induced Cre-mediated recombination. We performed olfactory manipulations on these animals, pulsing them with tamoxifen on the day in which the mice were first exposed to olfactory cues. After 6 weeks of either olfactory training or deprivation, we performed immunohistochemistry against Calb2, a marker for the n03-GC-1 cluster. We chose to analyze changes in the survival of this cluster because this population showed the most significant changes in cell distribution following experimental manipulations (Figure 5D). Interestingly, we did not detect changes in the total number of Calb2-positive cells within the granule cell layer across any the manipulations (Figure S3). This may be due to many of the cells in this population being stably integrated interneurons, which are thereby not affected by short-term olfactory manipulation. By examining co-localization of our lineage reporter and Calb2, we found that only 6.6% of adult-born granule cells differentiate into Calb2-positive granule cells (Figure S3). Comparing olfactory-deprived animals to naive animals, we observed that naris occlusion resulted in a 35% increase in the survival of Calb2-positive adult-born neurons (from 6.6% to 9% of adult-born neurons) (Figure S3). This observation is consistent with both the direction and relative intensity of cell distribution seen with experimental manipulations in single-cell-sequencing datasets (Figure 5). However, we did not observe changes between olfactory trained and naive animals (Figure S3). This is likely due to the scope of olfactory deprivation, which inherently affects the entire circuit, whereas olfactory training likely targets a smaller subset of neurons responsive to the odorants used. Notably, however, more sensitive cell-type-specific changes like this are indeed detectable within single-cell datasets of the entire bulb, which is an advantage of this methodology. Together, these data indicate that olfactory experience bidirectionally affects the developmental profile of adult-born neurons detailed by the pseudo-timeline analysis (Figure 5D) and reveals that each arm of the pseudo-timeline oppositely responds to activity.

## DISCUSSION

Here, we applied a high-throughput single-cell droplet-based RNA sequencing method to generate a transcriptome library for a mixture of 51,246 olfactory bulb cells that include neurons, astrocytes, oligodendrocyte lineages, immune cells, olfactory ensheathing cells, mesodermal cells, and blood vessel components. By analyzing transcripts enriched in these different populations, we uncovered cellular heterogeneity within each of these broadly characterized cell types. As a general reference, our study provides a list of selective markers that allow for future genetic labeling and cell-type-specific manipulations.

Inhibitory interneurons are major contributors to processing of neuronal circuit activity (Abraham et al., 2010; Lepousez and Lledo, 2013; Tan et al., 2010), yet interneuron heterogeneity has presented a major barrier toward fully understanding how these cells govern information processing. To better understand how interneurons contribute to circuit function, and since cellular complexity is established during development and maturation (Li et al., 2017), it is essential to uncover the mechanisms that regulate interneuron development within intact brain tissue. Recent studies that investigated the generation of cortical interneurons during embryonic development highlight the need for such investigations and have provided a great resource for understanding cortical interneuron complexity (Close et al., 2017; Mayer et al., 2018; Mi et al., 2018). Our data augment this important resource and reveal mechanisms that govern the maturation and drive heterogeneity of adult-born interneurons in the rodent olfactory system.

Continuous integration of adult-born neurons into existing brain circuits represents a notable source of circuit plasticity in the olfactory bulb (Nissant et al., 2009). Adult-born neurons exhibit a remarkable capacity to reorganize their own synaptic architecture as well as the structure and function of the surrounding network (Lledo and Saghatelyan, 2005; Ming and Song, 2011; Valley et al., 2013). Since the discovery of adult neurogenesis in the olfactory bulb, there has been a paucity of unbiased studies that have investigated the molecular changes of adult-born neurons throughout their development. Using a computational-based method of pseudo-timeline analysis, here we catalog an extensive list of genes that are differentially expressed throughout different stages of development, maturation, and integration of adult-born neurons. Additionally, by analyzing GRNs, we identified several transcription factors and putative effector combinations that potentially guide the integration and survival of adult-born neurons in a cell-type-specific manner. Our data reveal that *Sp3* and *Hdac2* are active GRNs in cell types located on a single axis (axis A) of a computed pseudo-timeline (Figure 4E). Axis-A-associated populations are increased following naris occlusion and reciprocally decreased after training (Figure 5D). Considering that *Sp3* and *Hdac2* function in programs associated with synaptic plasticity and neuronal survival, it is compelling to suggest that *Sp3* and *Hdac2* GRNs are differentially regulated by olfactory activity to drive survival of axis-A-specific cell clusters. In cells located on axis B of the pseudo-timeline, *Hivep2* and *Mef2d* regulons are highly active (Figure 4E). Although their role in olfactory bulb development is not well understood, these genes have been implicated in a variety of developmental and neurodegenerative disorders. For example, patients with mutations in *Hivep2* exhibit intellectual disability and dysmorphic features (Steinfeld et al., 2016). Myocyte enhancer factor 2 (MEF2) family transcription factors have been shown to be expressed in the developing mouse brain (Yamada et al., 2013) and have recently gained attention for their role in synaptic regulation and neuronal survival (Chan et al., 2014; Harrington et al., 2016; Mayer et al., 2018). Moreover, disruption of *Mef2d* and *Mef2c* pathways has been linked to amyotrophic lateral sclerosis (Arosio et al., 2016), and aberrant activation was shown to mediate neuronal death in mouse and fly models of Friedreich ataxia (Chen et al., 2016a, 2016b), indicating their potential role in neurodegeneration. Furthermore, Mef2d is thought to regulate neuronal gene expression in an activity-dependent manner through alternative polyadenylation site usage mechanisms (Flavell et al., 2008). Given this correlation, it is intriguing to consider that Hivep2 and Mef2d could potentially be involved in regulating adult-born neuron survival through mechanisms similar to those underlying neurodevelopmental and neurodegenerative diseases. Together, these data substantiate that our library of genes likely contains neurodevelopmental and neurodegenerative disease related genes and holds potential toward identifying disease- and/or cell-survival-related pathways.

Synaptic remodeling and circuit integration of adult-born neurons in the olfactory bulb are highly dependent on olfactory activity. Notably, increased numbers of cells within the immature neuronal cluster n04-immature following olfactory deprivation, and the reciprocal decrease in numbers observed with training, imply that adult-born neurons undergo a more rapid developmental transition in response to sensory enrichment (Figure 5D). Our previous studies (Quast et al., 2017) and others (Gheusi et al., 2000) showed that adult-born neurons integrate into circuits much more efficiently when they receive learned olfactory input. This implies a more rapid developmental transition from immature to mature granule cell states in response to olfactory enrichment. Interestingly, we have observed no change in clusters n05-PGC-2 (*Th*+), n09-GC-3 (*Rprm*+), and n12-GC-6 (*Tpbg*+). This finding matches what has been previously described, as n05-PGC-2 (*Th*+) and n12-GC-6 (*Tpbg*+) have been shown to be produced earlier in life and show minimal replacement through adult neurogenesis (Batista-Brito et al., 2008). Our data also reveal two populations of developing adult-born cells that respond oppositely to olfactory experience (Figure 5D, axis A and axis B). As such, the transcriptional signatures of cells in each group define two distinct cell populations that might be used to resolve cell-type-specific responses to olfactory activity.

Ultimately, these data provide a catalog of cellular heterogeneity in the mouse olfactory bulb. From this, we were able to discern molecular changes throughout maturation and integration of adult-born neurons. These data also reveal changes in the patterns of cell-type-specific specific gene expression in the olfactory bulb in response to olfactory enrichment and/or deprivation. Though these findings certainly reflect conserved features of adult olfactory bulb neurogenesis, we cannot rule out that these data may not represent a mechanistic continuum throughout adulthood and be generalizable across all stages of postnatal life. However, our study provides a valuable resource for identifying previously unknown markers that in turn will allow for the investigation of cell-type-specific contributions to circuit activity. Furthermore, the list of genes we revealed to be differentially regulated in adult-born neurons throughout development and maturation can be utilized in future studies to resolve the programs underlying the integration of new cells into existing brain tissue and will aid ongoing work toward understanding how such mechanisms function toward synapse formation, maintenance, and plasticity of adult brain circuits.

## STAR★METHODS

### CONTACT FOR REAGENT AND RESOURCE SHARING

Further information and requests for resources and reagents should be directed to the Lead Contact, Benjamin Arenkiel (arenkiel@bcm.edu).

### EXPERIMENTAL MODEL AND SUBJECT DETAILS

All animals used in this study were housed and handled according to US Department of Health and Human Services and Baylor College of Medicine IACUC guidelines. Both male and female C57BL/6NJ mice at 14 weeks of age were used for analyses, maintained on a 14-h light, 10-h dark cycle, with access to food and water *ad libitum*. Dlx1/2-CreER transgenic animals (Batista-Brito et al., 2008) were crossed to Rosa-LoxP-Stop-LoxP-TdTomato animals. 200mg/kg tamoxifen was administered by oral gavage at day 5 of olfac-tory training when odor presentation was started.

### METHOD DETAILS

#### Olfactory manipulation

For olfactory deprivation, naris occlusion was performed as described previously (Cummings et al., 1997). In brief, naris plugs were constructed by threading silk suture (Suture LOOK® Braided Silk Nonabsorbable Size 3–0 100 Yard Spool) through polyethylene (PE) tubing (Warner PE Tubing, PE-50/10 Cat: 64–0752). Hair was tied within a knot at one end of the suture, and was pulled into the tubing, generating an occlusion inside the tubing and a hair sticking out on one end of the tube. The opposite side of the tube was cut beveled for insertion into the nasal cavity. For increased olfactory activity, we implemented a previously described olfactory cued learning paradigm (Liu et al., 2017; Liu et al., 2018) where water deprived animals were trained to discriminate a water reward associated odor (S+) from a no reward associated (S−) odor. In brief, animals were trained to poke their nose into an odor delivery port to receive a water reward. Animals should choose to insert their nose into the side port when presented with the S+ odor and re-initiate the next trial by poking the center port when presented with the S- odor. If incorrect, they receive a 4 s time-out before being able to initiate the next trial. To ensure widespread circuit activation, we selected multiple pairs of similar odorants that each stimulate several distinct glomeruli. Odor pairs included anisole (Sigma 123226) and acetophenone (Sigma A10701), (S)-carvone (Sigma 22070) and (R)-carvone (Sigma 22060), (R)-limonene (Sigma 183164) and (S)-limonene (Sigma 62130), 1-pentanol (Sigma 138975) and 1-butanol (Sigma 281549), 1-heptanol (Sigma H2805) and 1-octanol (Sigma 95446), ethyl acetate (Sigma W241407) and methyl acetate (Sigma W267600), isoamylacetate (Sigma W205508) and amylacetate (Sigma W504009), 2,3-hexanedione (Sigma W255801) and 2-hexa-none (Sigma 02473) as Go and No-Go odorants, respectively. All odorants were mixed at 1% in mineral oil (Alfa Aesar 31911) when used separately, and mixed at 0.1% when used in a mixture. The 1% anisole and acetophenone pair was used for training. Mice were exposed to each odor pair for 2 days, and to mixtures for the rest of the task. Overall, olfactory deprivation and training was performed for 6 weeks to ensure robust circuit manipulation.

#### Tissue collection and dissociation

Animals were deeply anesthetized using isoflurane, and perfused intracardially with phosphate-buffered saline (PBS). Brains were dissected, and olfactory bulbs were removed from the rest of the brain. Since olfactory epithelium is juxtaposed to the olfactory bulb at the most anterior part of the brain, our data include some olfactory epithelium cells. Olfactory bulb tissue was dissociated according to the 10X Genomics Chromium sample preparation protocol. Briefly, tissue was cut into 1 mm^3^ pieces and placed in 2 mL papain solution (Worthington, PAPL) prepared according to manufacturer’s protocol in Hibernate E-Calcium (BrainBits, HECA100). Tissue was incubated in papain solution at 37°C for 20 min. Papain solution was removed and discarded by leaving the tissue at the bottom of the tube. 2 mL of HEB medium (BrainBits, HEB100) was added to the samples and triturated with a fire polished Pasteur pipette 10 – 15 times. Un-dissociated tissue was allowed to settle to the bottom of the tube for 1 min. Super-natant containing dissociated cells was moved to a different tube after passing through 70 μm mesh three times. Samples were centrifuged for 2 mins at 200 r*cf.* and the supernatant was discarded. Cells were resuspended in NbActiv1 (BrainBits, NbActiv1 100) solution and placed on ice for single cell RNA sequencing.

#### Single Cell RNA sequencing

Cells were counted and diluted in 1X PBS with 0.04% Bovine Serum Albumin (BSA) prior to loading onto the 10X Genomics Chromium instrument. Libraries were generated with the 10X Chromium Single Cell 3′ v2 reagent kit according to the manufacturer’s instructions, and sequenced on an Illumina Nextseq500.

#### Single cell RNA sequencing Analysis

Sequencing data was handled using the 10X Genomics Cell Ranger software (https://www.10xgenomics.com). Subsequently, expression matrices from each experiment were merged and imported into Seurat (version 2.2.1)(Butler et al., 2018), where Log normalization and scaling was performed (Thulasi Raman et al., 2016). The minimum gene per cell threshold was set to 200 for inclusion into the final digital expression matrix. Batch effects were corrected by regressing out the number of molecules per cell, mitochondrial genes, and identified with the RegressOut function (Seurat package). Principle components analysis (PCA) was performed and significant PCs were used as input for graph-based clustering. 2-dimensional visualization of the multi-dimensional dataset was done with t-SNE. Differential expression of the individual clusters was performed using the likelihood-ratio test for single cell gene expression (Seurat FindMarkers function, default parameters). To account for over-clustering, clusters that were not transcriptionally distinct were merged together into a single cluster. Doublets (2 different cell types within single droplet) were removed from the data-set. Gene Ontology (GO) analysis was performed with Metascape (http://www.metascape.org).

For pseudotemporal analysis, the normalized data from the indicated clusters calculated in Seurat was passed directly into Monocle2 (version 2.6.3) (Qiu et al., 2017). Next, we carried out density peak clustering (Monocle2 dpFeature procedure) to order cells based on the genes differentially expressed between clusters, with thresholds for density clustering set to 2 and 4 for rho and delta, respectively (Rodriguez and Laio, 2014). The top 1000 significant genes (ordered by qvalue) were used for ordering in all instances.

For gene regulatory network analysis, we generated co-expression networks via arboretum python software libraries (https://github.com/tmoerman/arboretum) and implemented GRNBoost2 (Friedman, 2002). For input into GRNBoost2 we used the processed and previously normalized data matrix extracted directly from Seurat. We then utilized the SCENIC package (version 0.1.7) to generate cell regulatory networks from our single-cell RNA sequencing data, with the mouse mm9 genome for cis-regulatory analysis (Aibar et al., 2017).

#### Stereotaxic injection and viral constructs

For stereotaxic injections, mice were anesthetized and maintained under anesthesia using vaporized isoflurane with O_2_. All injections were performed using a stereotaxic apparatus synced to Angle Two software. Targeting the core of the olfactory bulb (coordinates from Bregma in mm: ML 0.8, AP 4.5, DV −2.25), 690 nL of AAV-EF1α-DIO-EGFP-WPRE-hGHpA, serotype DJ/8 was injected as 10 pulses of 69 nl, 30 s apart, with the flow rate of 23 nl/s using a Drummond Nanoject II (Broomall, PA). Animals were sacrificed 14 days later for fluorescence imaging.

#### Immunohistochemistry

Animals were deeply anesthetized using isoflurane and were transcardially perfused with PBS followed by 4% Paraformaldehyde (PFA) (Diluted with 1XPBS from 16% Paraformaldehyde solution, EM grade, Electron microscopy sciences Cat:15710). Brains were dissected and post-fixed in 4% PFA at 4°C overnight. Brains were cryoprotected in 30% sucrose/PBS solution, embedded and frozen in OCT, and stored at −80°C. Tissue was cut coronally using a cryostat (Leica CM1860) at 40 μm directly into PBS. Free-floating sections were blocked for 1 h at room temperature in 10% normal goat serum blocking solution, made in PBS-T 0.3% (1 × PBS, 0.3% Triton X-100, pH 7.35). Sections were then incubated overnight at 4°C in a blocking solution containing primary antibodies at appropriate concentration (goat-anti-Omp at 1:10,000 Wako 544–10001, rabbit-anti-Calb2 at 1:500 Millipore AB5054, rabbit-anti-Th at 1:2000 Chemicon Ab152, rabbit-anti-Dcx at 1:400 Cell Signaling 4604, mouse-anti-NeuN at 1:500 Millipore MAB377, mouse-anti-Calb1 at 1:500 Abcam Ab9481, rabbit-anti-Sox11 at 1:500 Abcam Ab134107). The next day, sections were washed 4 times, 15 minutes each in PBS-T 0.1% (1 × PBS, 0.1% Triton X-100, pH 7.35), then incubated in blocking solution containing secondary antibody at 1:500 dilution (donkey-anti-goat-Cy3, goat-anti-rabbit Alexafluor-488 or goat-anti-mouse-Alexafluor-546) for 2 hours at room temperature. Sections were then washed 4 times for 15 minutes each in PBS-T 0.1%. All sections were mounted using DAPI Fluoromount-G (Southern Biotech, 0100–20). Detection of fluorescent expression was performed using a Leica TCS SPE confocal microscope under a 10 × or 20 × objective.

### QUANTIFICATION AND STATISTICAL ANALYSIS

We performed a Pearson’s chi-square test to determine if clusters were significantly different from the expected composition of a given condition (R base stats, chisq.test) (Xiao et al., 2018). The chisq.test was applied to each cluster using the totals from each condition to derive proper proportions. Calculated residuals or p values of residuals were then mapped to each cell within a cluster and visualized on the t-SNE. A p value of 0.05 was applied when assigning all discrete designations. For discrete scaling of the chisq.test, the continuous residual values were binned to a scale where clusters were designated as “up/increase” if > 0, “down/decrease” if < 0, and “none/no-change” if equal to zero. Thus, any cluster with a p value greater than 0.05 will have its residual set to 0.

### DATA AND SOFTWARE AVAILABILITY

All scripts used in this manuscript are available upon request. The accession number for the raw single-cell RNA sequencing data reported in this paper is GEO: GSE121891.

## Supplementary Material

1

2

3

4

5

6

## Figures and Tables

**Table:** KEY RESOURCES TABLE

REAGENT or RESOURCE	SOURCE	IDENTIFIER
Antibodies		
Goat polyclonal anti-Omp	Wako	Cat#544–10001
Rabbit polyclonal anti-Calb2	Millipore	Cat#AB5054
Rabbit polyclonal anti-Th	Chemicon	Cat#Ab152
Rabbit polyclonal anti-Dcx	Cell Signaling	Cat#4604
Mouse monoclonal anti-NeuN (clone A60)	Millipore	Cat#MAB377
Mouse monoclonal anti-Calb1 (clone CL-300)	Abcam	Cat#Ab9481
Rabbit monoclonal anti-Sox11 (clone EPR8192)	Abcam	Cat#Ab134107
Donkey anti-goat Cy3	Jackson ImmunoResearch	Cat#705-165-147
Goat anti-rabbit Alexafluor-488	Invitrogen	Cat#A-11034
Goat anti-mouse Alexafluor-546	Invitrogen	Cat#A-11030
Bacterial and Virus Strains		
pAAV-EF1 α-DIO-EGFP-WPRE-hGHpA	Neuroconnectivity Core at Baylor College of Medicine	N/A
Deposited Data		
FastQ files	This Paper	GEO: GSE121891
Compiled gene expression matrices	This Paper	GEO: GSE121891
Experimental Models: Organisms/Strains		
Mouse: C57BL/6NJ	The Center for Comparative Medicine (CCM) at Baylor College of Medicine	N/A
Mouse: Dlx1/2-CreER: Tg(I12b-cre/ERT2,-ALPP)37Fsh/J	Batista-Brito et al., 2008	N/A
Mouse: Rosa-LoxP-Stop-LoxP-TdTomato:B6.Cg- Gt(ROSA)26Sortm14(CAG-tdTomato)Hze/J	The Jackson Laboratory	JAX: 007914
Software and Algorithms		
Seurat	Butler et al., 2018	https://github.com/satijalab/seurat/tree/develop
Monocle 2	Trapnell et al., 2014	https://github.com/cole-trapnell-lab/monocle-release
SCENIC	Aibar et al., 2017	https://github.com/aertslab/SCENIC
Arboretum python software libraries	Friedman 2002	https://github.com/tmoerman/arboretum
10x cellranger 2.0.0	10x Genomics	https://support.10xgenomics.com/single-cell/software/overview/welcome
Other		

## Highlights

- Single-cell sequencing reveals cellular heterogeneity in the mouse olfactory bulb
- Differential gene expression uncovers selective markers for cell types
- Pseudotemporal ordering of adult-born neurons reveals developmentally governed genes
- Olfactory experience changes the cellular composition of olfactory bulb circuits
