# Supplementary material for: Single-Cell RNA-Seq of Mouse Olfactory Bulb Reveals Cellular Heterogeneity and Activity-Dependent Molecular Census of Adult-Born Neurons
Source: Cell Rep. Author manuscript; Available in PMC 2019 Jan 22. (PMC6342206; doi:10.1016/j.celrep.2018.11.034)
Supplement: 1 [file NIHMS1516652-supplement-1.pdf]

Cell Reports, Volume *25*

Supplemental Information

Single-Cell RNA-Seq of Mouse Olfactory Bulb Reveals Cellular Heterogeneity and Activity-Dependent Molecular Census of Adult-Born Neurons

Burak Tepe, Matthew C. Hill, Brandon T. Pekarek, Patrick J. Hunt, Thomas J. Martin, James F. Martin, and Benjamin R. Arenkiel

A

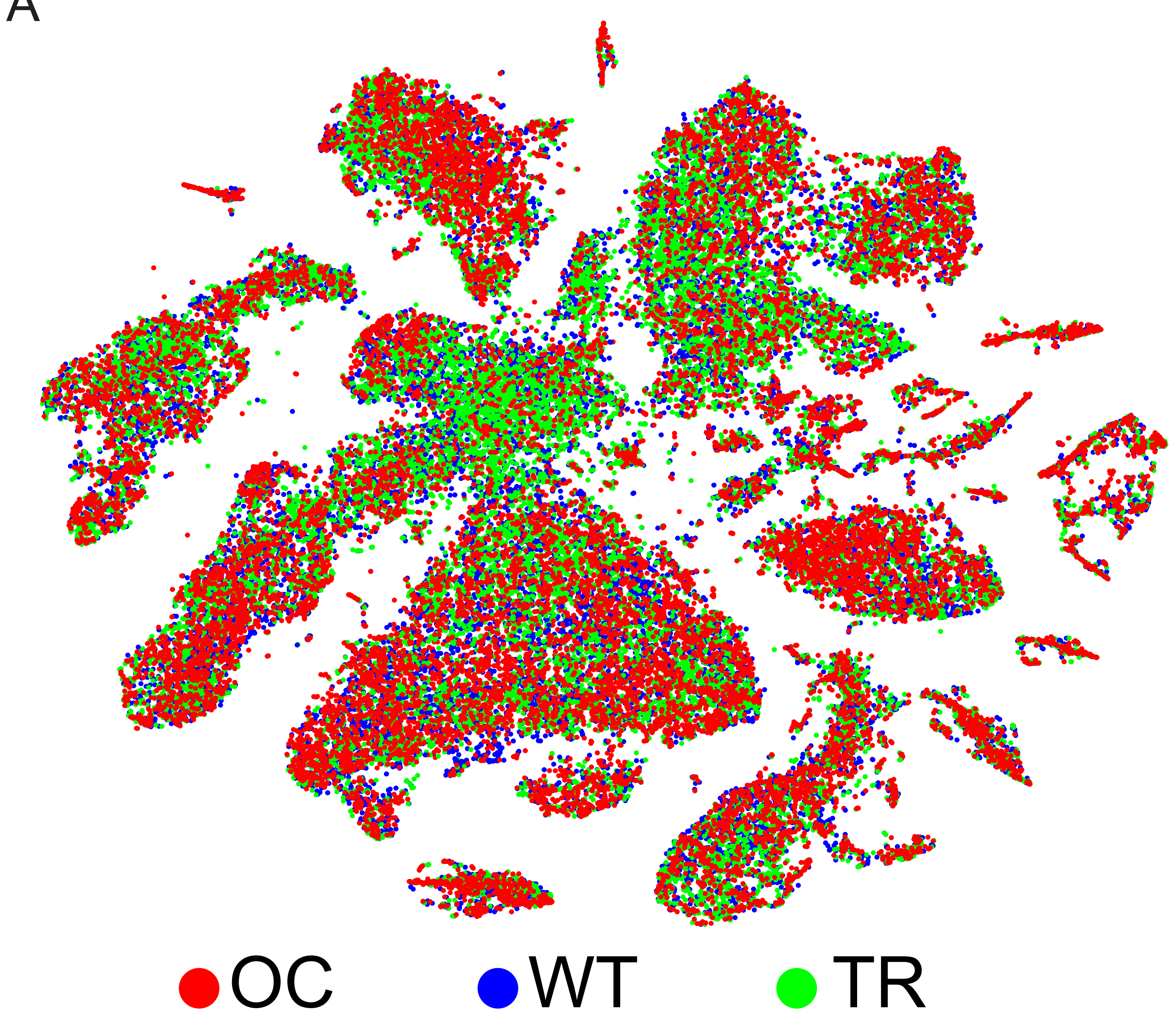

B

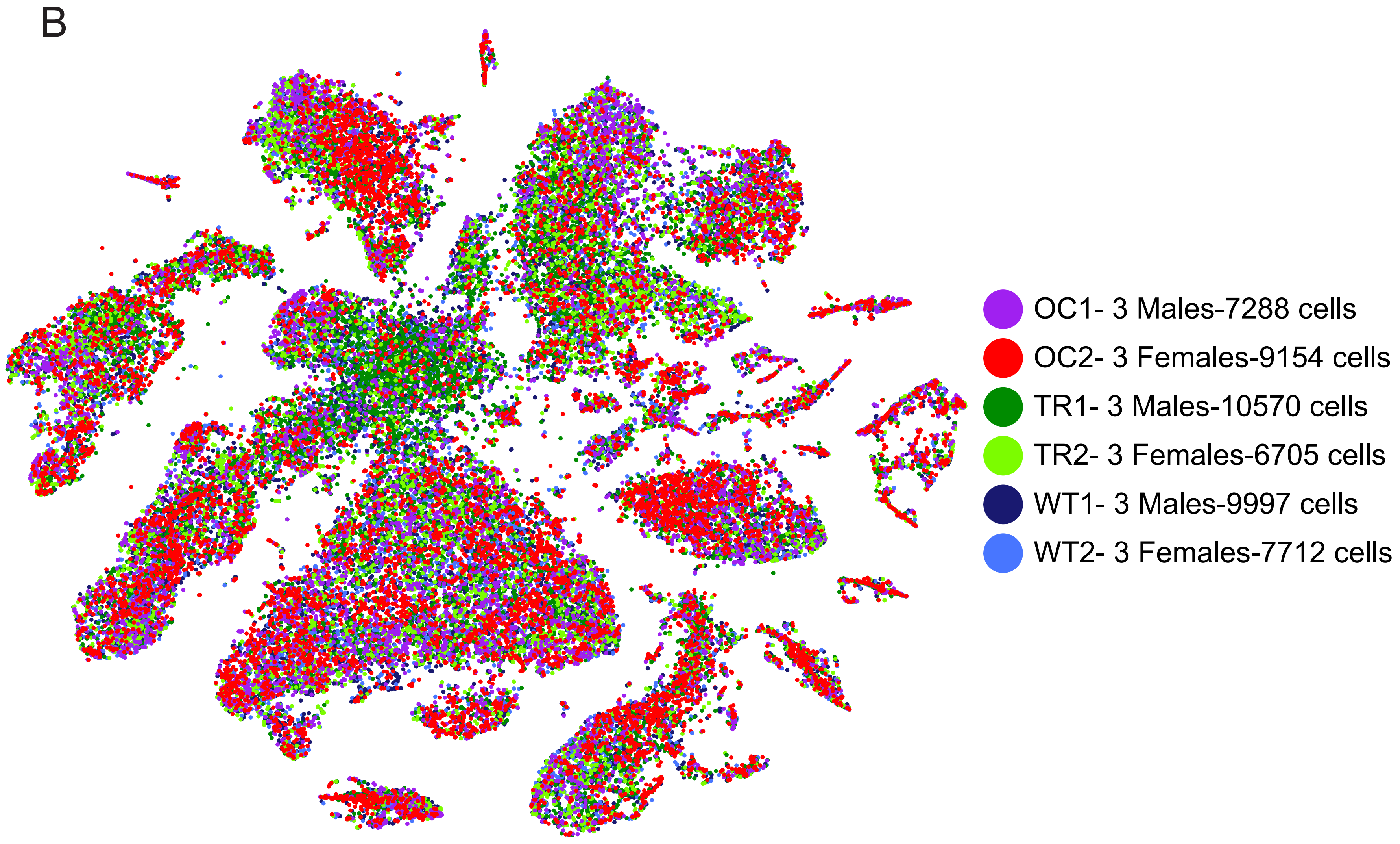

C

	OC1	OC2	TR1	TR2	WT1	WT2	Total
a01.RBCs	54	30	21	11	80	23	219
a02.MyOligo	135	224	111	94	167	127	858
a03.Neuron01	185	106	231	138	245	184	1089
a04.Mono	81	127	85	92	137	101	623
a05.MicroG1	254	386	509	345	410	356	2260
a06.MicroG2	117	177	105	136	117	138	790
a07.MicroG3	102	152	252	151	148	146	951
a08.Mf	106	108	91	70	110	87	572
a09.EC1	296	508	494	329	460	437	2524
a10.EC2	120	216	245	98	238	207	1124
a11.Mural1	23	42	39	11	22	21	158
a12.Mural2	87	174	127	109	146	126	769
a13.Neuron02	67	36	48	33	27	33	244
a14.Neuron03	110	105	148	88	233	177	861
a15.Neuron04	524	725	469	267	604	385	2974
a16.Neuron05	286	378	400	258	392	229	1943
a17.Neuron06	563	336	737	539	677	483	3335
a18.Neuron07	41	34	221	91	173	104	664
a19.Neuron08	188	280	593	384	388	365	2198
a20.Neuron09	106	133	186	194	165	167	951
a21.Neuron10	160	218	148	107	208	172	1013
a22.Neuron11	18	31	40	16	33	30	168
a23.Neuron12	61	69	117	74	93	59	473
a24.Neuron13	477	840	563	470	607	354	3311
a25.Neuron14	23	26	22	28	31	11	141
a26.Astro1	484	654	520	385	629	458	3130
a27.Astro2	214	200	533	234	321	278	1780
a28.Astro3	57	82	33	35	73	56	336
a29.Mes1	39	48	54	24	33	32	230
a30.OEC1	408	523	233	276	476	336	2252
a31.Neuron15	9	22	10	5	17	5	68
a32.OEC2	135	229	136	102	162	119	883
a33.OEC3	573	702	602	476	585	498	3436
a34.OEC4	459	530	712	466	550	523	3240
a35.OEC5	298	295	255	228	370	294	1740
a36.Mes2	125	121	135	72	121	79	653
a37.Neuron16	255	210	1260	219	677	447	3068
a38.OPC	48	77	85	50	72	65	397
Total	7288	9154	10570	6705	9997	7712	

Figure S1. Single cell transcriptome analysis delineates mouse olfactory bulb cellular heterogeneity, Related to Figure 1.
A) The entire cellular composition is visualized using the graph-based clustering method, t-SNE. Individual single-cell transcriptomes are colored according to experimental group. OC: naris occluded, WT: wildtype, TR: olfactory trained.
B) The entire cellular composition is visualized using t-SNE. Individual single-cell transcriptomes are colored according to the experimental batch, the number of animals pooled, and the number of cells recovered from that batch.
C) Table of the number of cells recovered for each cell cluster per sequencing batch.

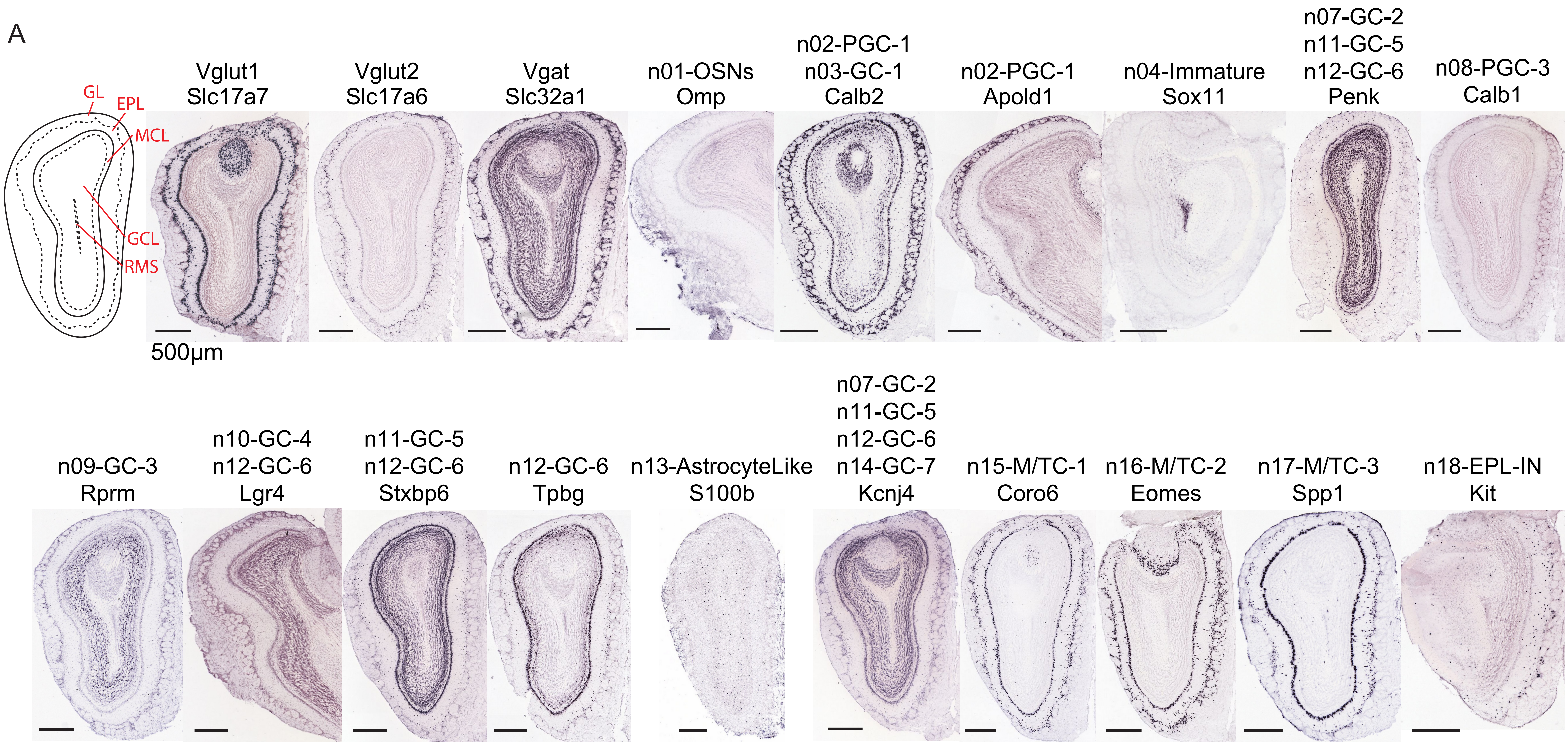

Figure S2. Unsupervised transcriptome based clustering reveals unknown neuronal subtypes, Related to Figure 2.

A) Allen brain atlas in situ hybridization data corresponding to the most highly enriched cluster markers. Top left image shows a schematic representation of OB architecture. GL: Glomerular layer, EPL: External plexiform layer, MCL: Mitral cell layer, GCL: Granule cell layer, RMS: Rostral migratory stream, scale bar is 500 µm.

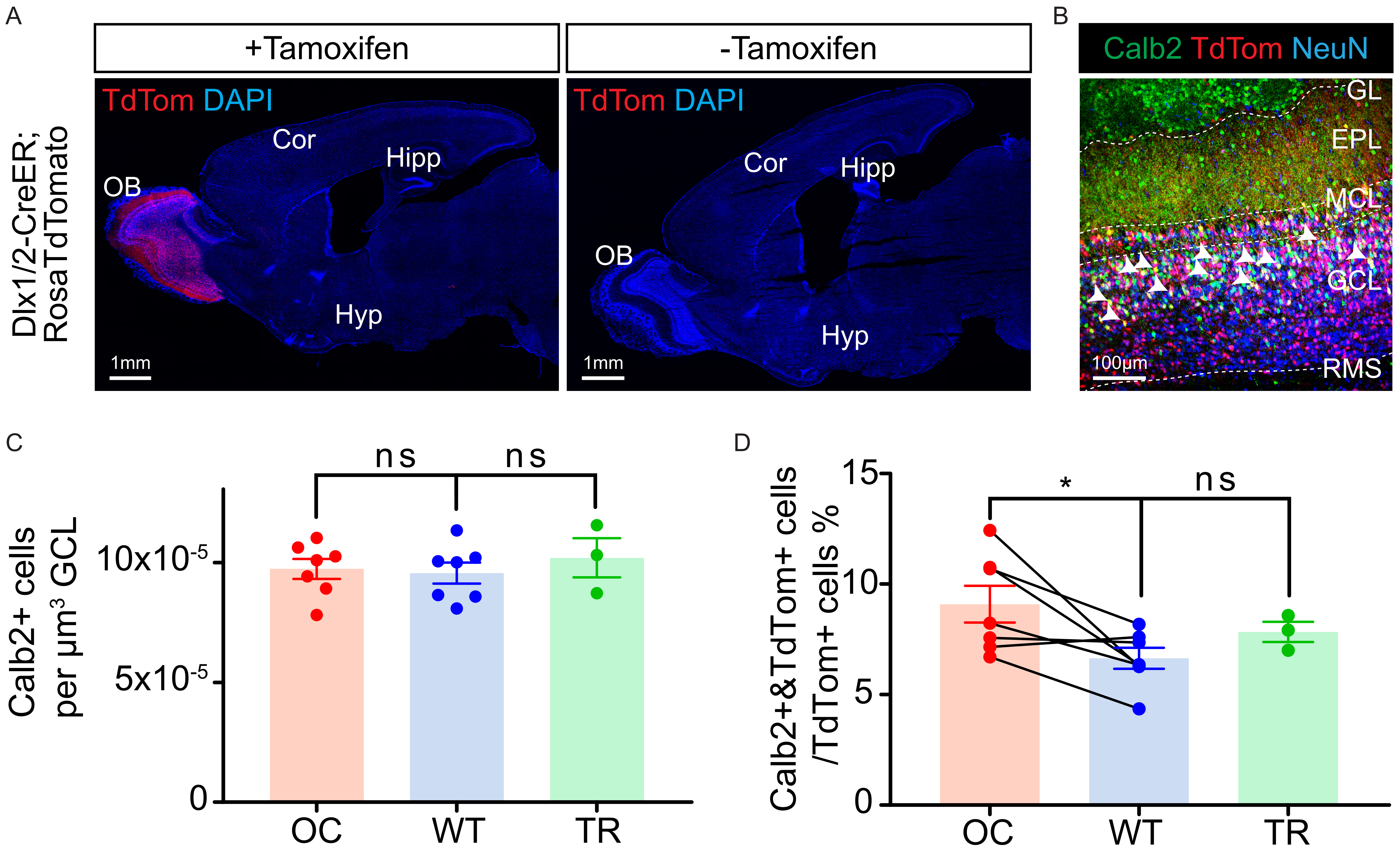

Figure S3. Olfaction alters tissue composition by selective addition of adult-born interneuron subtypes, Related to Figure 5.

A) Dlx1/2-CreER; Rosa-LoxP-Stop-LoxP-TdTomato transgenic animals pulsed with Tamoxifen. Dlx1/2-positive cells are labeled in the OB in animals pulsed with Tamoxifen (left panel) and are not labeled in Dlx1/2-CreER; Rosa-LoxP-Stop-LoxP-TdTomato transgenic animals that were not pulsed with tamoxifen (right panel). OB: Olfactory bulb, Cor: Cerebral Cortex, Hipp: Hippocampus, Hyp: Hypothalamus. Scale bar is 1 mm.

B) Representative coronal OB image from tamoxifen-pulsed Dlx1/2-CreER; Rosa-LoxP-Stop-LoxP-TdTomato transgenic animals stained with α-Calb2 and α-NeuN. These images were used for quantification of adult-born neuron differentiation. Scale bar is 100 um.

C) Calb2-positive cell density in granule cell layer of olfactory manipulated animals. OC: naris occlusion, WT: wildtype naïve, TR: olfactory trained.

D) Percentage of adult-born neurons that differentiated into Calb2-positive fate after olfactory manipulation. OC: naris occlusion, WT: wildtype naïve, TR: olfactory trained. $^{*}p < 0.05$.
